# Expansion of permanent first molars with rapid maxillary expansion appliance anchored on primary second molars

**DOI:** 10.4317/jced.54585

**Published:** 2018-03-01

**Authors:** Michele Tepedino, Maciej Iancu-Potrubacz, Domenico Ciavarella, Francesco Masedu, Laura Marchione, Claudio Chimenti

**Affiliations:** 1 Department of Biotechnological and Applied Clinical Sciences, University of L’Aquila, L’Aquila, Italy; 2 Department of Clinical and Experimental Medicine, University of Foggia, Foggia, Italy

## Abstract

**Background:**

To evaluate how the amount of expansion of the primary second molars, the patient’s age, and the skeletal maturation stage influence the amount of expansion at the level of the permanent first molars.

**Material and Methods:**

Fifty-five patients aged between 6 and 11 years with a cervical vertebral maturation stage of CS1 or CS2 were retrospectively selected. The intermolar width was measured before and after expansion to evaluate the amount of expansion achieved at the level of the primary second molars and the permanent first molars. Stepwise multiple linear regression was used to evaluate how the amount of primary molars expansion, the patient’s age, and the cervical vertebral maturation stage predict the amount of permanent molar expansion.

**Results:**

A significant regression equation was found, and for every 1 mm of primary molar expansion, 0.91 mm of permanent molar expansion can be expected. An age between 6 and 11 years and the CS1 or CS2 skeletal maturation stage were not significant predictors of permanent molar expansion.

**Conclusions:**

A rapid maxillary expansion appliance anchored on primary second molars is effective in expanding the permanent molars to correct a transverse maxillary deficiency in prepubertal patients, transferring the risks associated with the large forces used to the primary teeth.

** Key words:**Maxillary expansion, transversal deficiency, primary molars.

## Introduction

Maxillary transverse deficiency is a relatively common feature and can lead to a posterior unilateral or bilateral crossbite. The prevalence of posterior crossbite in a southern Italian population was reported to be around 14% (1). The treatment of choice for this malocclusion is rapid maxillary expansion (RME), which has proved to be effective in correcting the transverse maxillary deficiency, coordinating the maxillary and mandibular arches, and recovering space in the anterior section of the maxillary arch in patients with a tooth size–arch length discrepancy (2-5). Studies have also proved that the results achieved with this type of treatment are relatively stable in the long term (6,7). The best timing for this kind of treatment is in the prepubertal stage (8). Studies on autoptic material have revealed that before the age of 10 the maxillary suture is broad and smooth, whereas in later stages of growth it develops more interdigitations, presenting greater resistance to opening (9,10).

The RME appliance is usually made of an expansion screw connected through stiff metal arms to bands for the upper first molars, and sometimes also for the upper first premolars. The anchorage teeth experience a buccal tipping of mean 5° during expansion (2) that partially relapses during the retention phase. In addition, the forces produced during RME can be heavy (11,12), and some iatrogenic effects can be observed on the anchorage teeth like exostosis, pulp stones, root resorption, and periodontal damage (13-15). For these reasons, some authors suggest using the primary second molars as anchorage for the RME appliance. The aim of the present study was therefore to evaluate the amount of expansion at the level of the permanent first molars in relation to the amount of expansion on the primary second molars, and if age and skeletal maturation influence this amount. The null hypothesis was that the amount of expansion of primary molars, the patient’s age, and skeletal maturation have no effect on the amount of expansion of permanent molars.

## Material and Methods

The records of patients treated with RME at the Orthodontic Department of the University of L’Aquila from January 2013 to September 2017 were screened for the following inclusion criteria:

-Age between 6 and 11 years

-Prepubertal stage of growth assessed through the cervical vertebral maturation (CVM) method (16) between CS1 and CS3

-Treatment with an RME appliance with two bands bonded on the primary second molars

-Intra-oral photographs recorded before and after the active expansion phase

-High quality of occlusal photographs of the upper arch, which should have been perfectly perpendicular to the occlusal plane.

**Figure 1:**
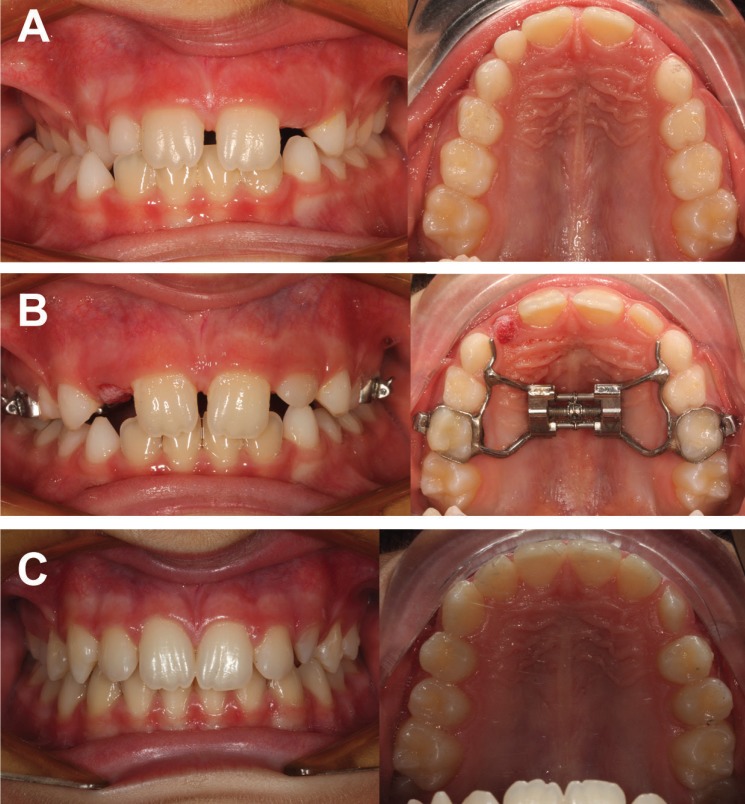
Example of a case with unilateral posterior crossbite successfully treated with a RME appliance anchored to primary second molars. A, pre-treatment intraoral photographs; B, after expansion with the RME appliance in place; C, post-treatment intraoral photographs after debonding of the appliance, showing the successful treatment of the posterior crossbite.

**Figure 2:**
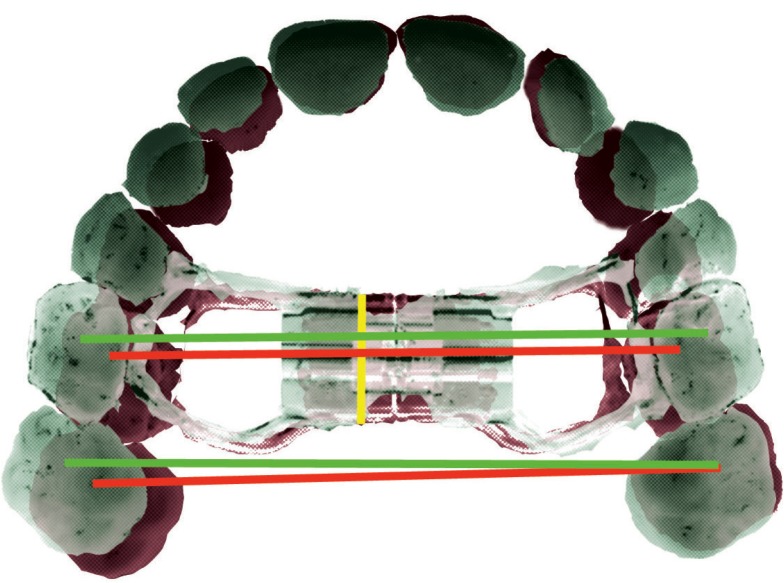
Schematic representation of intermolar width changes measured at T0 and T1. Yellow line, measurement of antero-posterior width of the expansion screw to calibrate the images; red line, measurement of T0 width of primary second molars and permanent first molars; green line, measurement of T1 width of primary second molars and permanent first molars.

Intra-oral upper occlusal photographs before (T0) and after (T1) the active expansion phase were collected for all the subjects that were eligible for inclusion in the study group. RME treatment was performed using a Hyrax-type appliance with two bands for left and right primary second molars, and no connection to the permanent molars (Fig. 1). The type of expansion screw used was determined, and then technical information about the physical screw dimensions was retrieved from each manufacturer. Occlusal photographs were imported into ImageJ software (US National Institutes of Health, Bethesda, Maryland, USA) (17), and the antero-posterior width of the body of the expansion screw was used as a reference to calibrate the images. The arch width was measured between the primary second molars and the permanent first molars at both T0 and T1 using the central sulcus of the occlusal face as a reference for the measurements (Fig. 2). All measurements were performed by the same well-trained operator (MT). Lateral cephalograms were used to assess the CVM staging independently by two operators (MT and MIP).

Sample size calculation to perform a linear regression with a standard deviation of permanent molar expansion of 3, an estimated correlation coefficient of 0.4, and a slope estimate obtained from regressing deciduous molar expansion against a permanent molar expansion of 0.9 — as retrieved from a pilot study — revealed that 43 subjects would be needed to be able to reject the null hypothesis that this slope equals zero with a power of 0.8 and a type I error probability of 0.05.

-Error of the method

Twenty-five subjects out of the entire study group were randomly selected using an online tool (www.randomizer.org), and the measurements were repeated by the same observer after a two-week interval. The two sets of repeated measurements were used to calculate the standard error using the Dahlberg formula (s=√(Sd^2 )/2n, where d= difference between the first and second measurements). Bland–Altman plots were used to check for intra-observer reliability between the two sets of measurements (18). Regarding the CVM staging assessment, inter-rater agreement between the two reviewers (MT and MIP) was calculated using Cohen’s kappa statistics.

-Statistical analysis

Descriptive statistics were performed for all the variables. A stepwise multiple linear regression was calculated to predict the amount of permanent first molar expansion based on the amount of primary second molar expansion, adjusting for patient’s age and CVM stage. Stepwise criteria were set with a probability to enter the model of F<= 0.05, and a probability to be removed of F>= 0.1. Normal P-P Plots were also used to check the assumption of homoscedasticity and normality of residuals. First-type error was set as 0.05. Statistical analysis was carried out using SPSS software (SPSS for Windows, Version 13.0. Chicago, SPSS Inc.).

## Results

**Figure 3:**
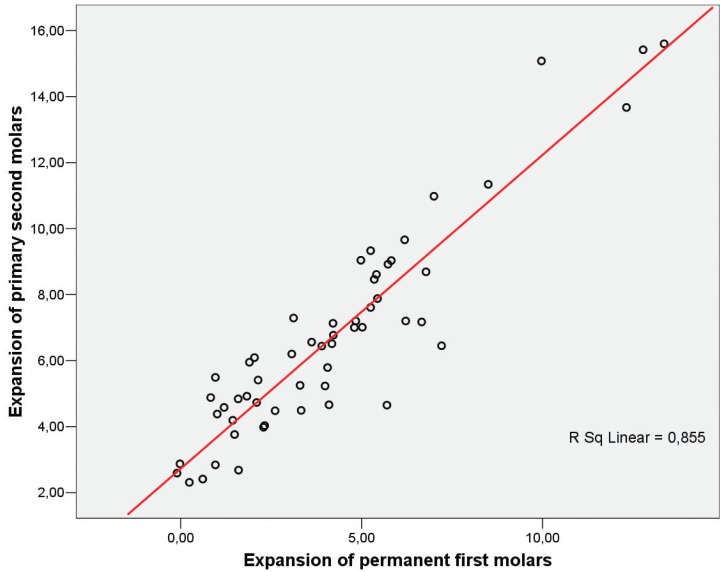
Scatterplot of permanent first molar expansion (X axis) and primary second molars expansion (Y axis).

Fifty-five consecutively treated patients who met the inclusion criteria were enrolled in the study group (F= 22; M= 33). The mean age was 8.4 ± 1.1, with a range of 6.0 to 11.0 years, and all subjects had a CVM stage of CS1 or CS2 ( Table 1). Descriptive statistics for primary and permanent molar width and expansion are reported in  Table 2. The stepwise regression procedure led to the removal of the variables of age and CVM stage, leaving only the amount of primary second molar expansion in the final model. A significant regression equation was found (F[1, 53] = 379.29, *p*< 0.001), with an adjusted R2 of 0.875 (Fig. 3,  Table 3, Table 4). Predicted permanent molar expansion was equal to -1.99 + 0.91 (primary molar expansion), where primary molar expansion is measured in mm; therefore, permanent molar expansion increased by 0.91 mm for each 1 mm of primary molar expansion. Primary molar expansion was a significant predictor of permanent molar expansion (*p*< 0.001).

**Table 1:**
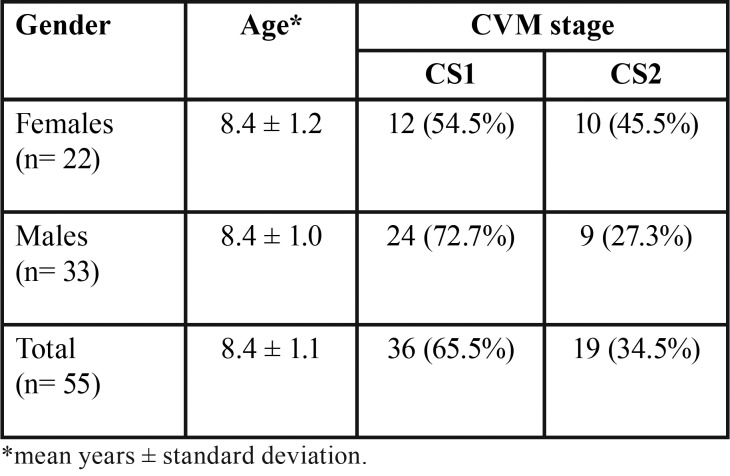
Composition and baseline characteristics of the study sample.

**Table 2:**
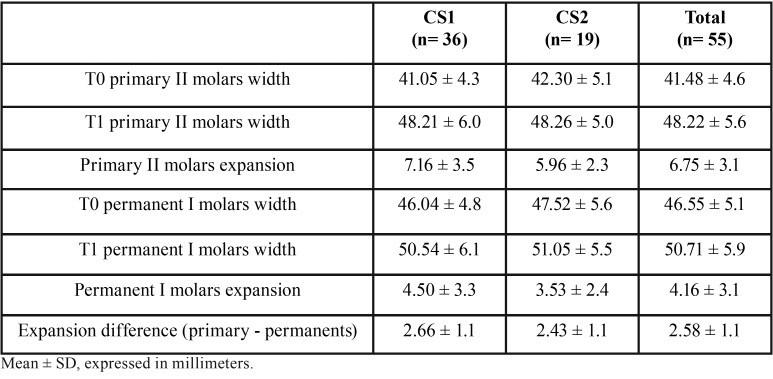
Descriptive statistics for molar width and expansion measurements.

**Table 3:**
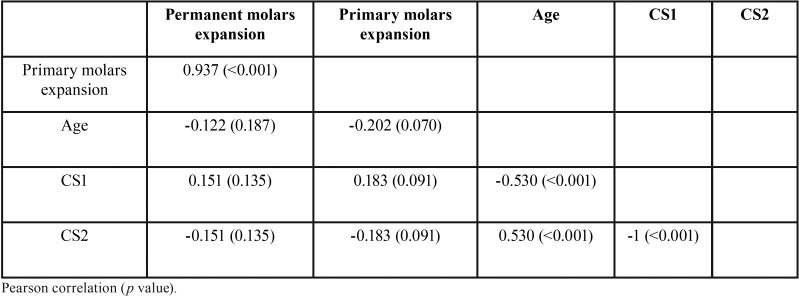
Correlations matrixes for all the studied variables.

**Table 4:**
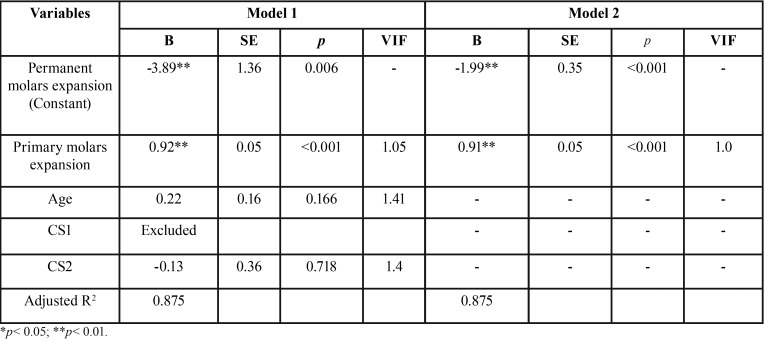
Stepwise multiple linear regression outcome.

Regarding the error of the method for expansion measurements, the standard error calculated with Dahlberg’s formula was 0.3 mm for both the outcome variables (primary second molar expansion and permanent first molar expansion), while the Bland–Altman plots revealed no systematic errors. Regarding the assessment of CVM stage, inter-rater agreement between the reviewers was good, with a Cohen’s kappa value of 0.857 (*p*<0.001).

## Discussion

The aim of the present study was to estimate the amount of permanent first molar expansion that can be achieved when the RME appliance is applied to the primary second molars. The results demonstrate that for every 1 mm of expansion at the level of the primary second molars, 0.91 mm of expansion should be expected at the level of the permanent first molars.

The rationale behind the decision to apply the RME appliance to the primary molars is to transfer the forces produced during the maxillary expansion to deciduous teeth instead of permanent teeth, and to reduce the risk of an iatrogenic scissor bite. Even though some authors have demonstrated that there is generally good functional adaptation to RME treatment (19,20), the forces produced during rapid expansion of the maxillary suture can be extremely high and exceed 100 N (21); those forces are absorbed by all the circummaxillary sutures (22) and can produce strains also at the level of the cranial base, leading sometimes to unwanted side effects (23,24). The teeth on which the appliance is bonded also experience these high force levels, which can lead to several complications: exostosis, pulp stones, root resorption, and periodontal damage are the most frequent (13-15). In addition, the anchorage teeth always experience a certain amount of buccal tipping, even if the RME appliance is correctly designed, with stiff connectors and the screw positioned as close as possible to the palatal vault, which is the best biomechanical configuration to maximise the skeletal effects (25). McNamara *et al.* (2003) measured this amount of buccal tipping and showed a mean of 5°; this amount of buccal tipping is generally unwanted, because it reduces the amount of skeletal expansion that can be achieved. To reduce the risk of complications and maximise the gain in skeletal transverse dimension with the minimum dental compensatory effect, the RME treatment should be performed before the pubertal growth spurt (8); in fact, Melsen and Melsen (1982) demonstrated that before the age of 10 the sagittal maxillary suture offers the lowest resistance to opening. Treating patients during the early mixed dentition phase also has the advantage of increasing the intercanine width, thus relieving anterior crowding and promoting at an early stage the development of the arches towards a wider archform, creating better conditions for harmonic growth (26). On the other hand, treating patients too early, in the deciduous dentition stage, has less clinical advantage, because in 34% of cases the first permanent molars will erupt in crossbite even after transverse dimension correction (27). During the early mixed dentition developmental stage, the degree of root resorption of the primary second molars allows them to be used as anchorage for an RME appliance. Indeed, Ugolini *et al.* (28) showed that until the length of the roots equals the length of the crown, those teeth are able to withstand the orthopaedic forces induced by the appliance for the required treatment time. In this way, all the possible side effects are transferred to teeth that will exfoliate later. It was demonstrated that an RME appliance anchored on primary second molars is effective in correcting a crossbite at the level of the permanent first molars (29). Moreover, this correction was showed to be stable at least 2.4 years post-treatment, and only 10% of the permanent molar expansion was lost during the post-retention phase (29). This observation can be related to the fact that the expansion at the level of the permanent molars is due only to skeletal effects, and since those teeth are not touched directly by the appliance, they experience no buccal tipping (28). The stability of the permanent molars can be explained by the ‘funnel theory’ described by Van Der Linden (30). Since those molars are not bound to the appliance, when they reach a normal position they are maintained by occlusion in a stable relationship; furthermore, the relapse of the increase in the transverse dimension is partially explained by the buccal tipping of the teeth after RME that rapidly relapses in the post-retention phase (31). When the RME is anchored on primary second molars, a larger and more stable expansion of the intercanine width is also observed, compared to the use of an RME anchored on permanent molars and probably caused by the more anterior position of the screw (28), which represents an advantage in terms of space recovery in the anterior section of the upper arch. Because the permanent molars are free to settle into the new occlusion, a certain amount of distorotation is also observed, which cannot happen if the RME is anchored to the permanent teeth (32).

The results of the present study suggest that a certain amount of overcorrection of the primary second molar expansion is needed to achieve the desired expansion of the permanent first molars, and this amount can be calculated from the following regression equation:

Permanent molar expansion(predicted)= B0 + (0.91*∆ primary molar expansion)

Age and CVM stage were not significant predictors of permanent molar expansion: since all patients were in a mixed dentition stage, they had a similar age (with a five-year range) and were all in the CS1 or CS2 stage of skeletal maturation, which are both prepubertal stages. Therefore, from the results of the present study, there was no difference in the prediction of permanent molar expansion if the patients were in either the CS1 or CS2 stage of vertebral maturation.

The measurements recorded in the present study were demonstrated to be reliable by the small error of the method, and the use of photographs or scanned images for this kind of measurement was already validated by other authors (26,33,34). The main limitation of the present study was its retrospective design, but care was taken during the case selection process by rigidly following a time interval criterion to select only consecutive cases, thus reducing the risk of selection bias.

## Conclusions

For every 1 mm of primary second molar expansion, 0.91 mm of permanent first molar expansion can be predicted. The patient’s age between 6 and 11 years old and a CVM stage of CS1 or CS2 were not significant predictors of the amount of permanent first molar expansion. An RME appliance anchored to the primary second molars is effective in expanding the permanent first molars and helps reduce the risk of complications for the permanent teeth that are usually related to this kind of treatment.
